# Surface mechanomyography and electromyography provide non-invasive indices of inspiratory muscle force and activation in healthy subjects

**DOI:** 10.1038/s41598-018-35024-z

**Published:** 2018-11-16

**Authors:** Manuel Lozano-García, Leonardo Sarlabous, John Moxham, Gerrard F. Rafferty, Abel Torres, Raimon Jané, Caroline J. Jolley

**Affiliations:** 1 Biomedical Signal Processing and Interpretation group, Institute for Bioengineering of Catalonia (IBEC), The Barcelona Institute of Science and Technology (BIST), Barcelona, Spain; 2 Biomedical Research Networking Centre in Bioengineering, Biomaterials and Nanomedicine (CIBER-BBN), Barcelona, Spain; 3 Department of Automatic Control (ESAII), Universitat Politècnica de Catalunya (UPC)-Barcelona Tech, Barcelona, Spain; 4 Faculty of Life Sciences & Medicine, King’s College London, King’s Health Partners, London, United Kingdom; 5 King’s College Hospital NHS Foundation Trust, King’s Health Partners, London, United Kingdom; 6 Centre for Human & Applied Physiological Sciences, School of Basic & Medical Biosciences, Faculty of Life Sciences & Medicine, King’s College London, King’s Health Partners, London, United Kingdom

## Abstract

The current gold standard assessment of human inspiratory muscle function involves using invasive measures of transdiaphragmatic pressure (P_di_) or crural diaphragm electromyography (oesEMG_di_). Mechanomyography is a non-invasive measure of muscle vibration associated with muscle contraction. Surface electromyogram and mechanomyogram, recorded transcutaneously using sensors placed over the lower intercostal spaces (sEMG_lic_ and sMMG_lic_ respectively), have been proposed to provide non-invasive indices of inspiratory muscle activation, but have not been directly compared to gold standard P_di_ and oesEMG_di_ measures during voluntary respiratory manoeuvres. To validate the non-invasive techniques, the relationships between P_di_ and sMMG_lic_, and between oesEMG_di_ and sEMG_lic_ were measured simultaneously in 12 healthy subjects during an incremental inspiratory threshold loading protocol. Myographic signals were analysed using fixed sample entropy (fSampEn), which is less influenced by cardiac artefacts than conventional root mean square. Strong correlations were observed between: mean P_di_ and mean fSampEn |sMMG_lic_| (left, 0.76; right, 0.81), the time-integrals of the P_di_ and fSampEn |sMMG_lic_| (left, 0.78; right, 0.83), and mean fSampEn oesEMG_di_ and mean fSampEn sEMG_lic_ (left, 0.84; right, 0.83). These findings suggest that sMMG_lic_ and sEMG_lic_ could provide useful non-invasive alternatives to P_di_ and oesEMG_di_ for the assessment of inspiratory muscle function in health and disease.

## Introduction

The ability to measure respiratory muscle function is clinically important in the assessment of neuromuscular^1^ and respiratory disease^2^. In the respiratory system, respiratory muscle force is usually estimated as pressure, and shortening as lung volume change or displacement of chest wall structures^3^. Moreover, respiratory muscle force is tightly related to the level of activation of the muscles^3,4^, which can be assessed by electromyography. The diaphragm is the main inspiratory muscle^5,6^, and so it is important to be able to measure diaphragm function accurately. Accurate assessment of diaphragm pressure generation and activation, however, requires invasive procedures, such as the balloon-catheter technique, to measure transdiaphragmatic pressure (P_di_)^7^, or crural diaphragm electromyography using a multipair oesophageal electrode (oesEMG_di_)^2,8–10^. These invasive tests can be uncomfortable for patients and require some skill from the operator involved. They are therefore rarely carried out in clinical practice. The development of novel, non-invasive indices of diaphragm force output and activation would therefore represent a significant advance in the assessment of patients with disordered respiratory muscle function.

Transcutaneous assessment of muscle fibre vibration during contraction provides the opportunity to non-invasively quantify an index of the force exerted by the underlying contracting skeletal muscle^11^. In common with other skeletal muscles^11^, diaphragm muscle fibres vibrate laterally during contraction^12^. These vibrations, related to the mechanical activation of diaphragm muscle fibres, can be non-invasively recorded as sound, using microphones (phonomyography), to provide a non-invasive index of electromechanical coupling, recruitment, and P_di_ generation by the diaphragm^12^. Alternatively, inspiratory muscle fibre vibration can be recorded by mechanomyography, using skin-surface accelerometers positioned on the chest wall over the lower intercostal spaces, proximal to the zone of apposition of the diaphragm (sMMG_lic_)^13–15^. The surface mechanomyogram (sMMG) is considered to be the mechanical counterpart of motor unit (MU) electrical activity as measured by surface electromyography (sEMG)^16^, and confers several important advantages. Unlike sEMG, sMMG is not influenced by skin preparation, bioelectrical interference from other muscles or by power line interference. Despite this, the use of sMMG to assess inspiratory muscle function has been relatively unexplored, and sMMG_lic_ has not been compared with gold standard invasive measures of P_di_, during voluntary respiratory manoeuvres.

Measurements of both sEMG_lic_ and sMMG_lic_ are, however, contaminated by cardiac artefacts, especially using the average rectified value or root mean square (RMS) as standard. Recently, fixed sample entropy (fSampEn) has been proposed as a means to estimate the respiratory muscle effort from sMMG_lic_^13,14^ and sEMG_lic_^17^ signals, with less interference from cardiac artefacts. However, fSampEn has not previously been applied to analysis of gold standard invasive oesEMG_di_ measures.

The principal aim of the study was, therefore, to investigate the use of sMMG_lic_ as a novel non-invasive index of inspiratory muscle force, by examining its relationship with the invasive gold standard, P_di_, in healthy subjects during an incremental inspiratory threshold loading protocol. We hypothesized that there would be close positive linear relationships between mean fSampEn |sMMG_lic_| and mean P_di_, and between the time-integrals of these signals. The relationship between invasive oesEMG_di_ and non-invasive sEMG_lic_ was also investigated. In addition, we aimed to compare the utility of RMS- and fSampEn-based techniques to analyse oesEMG_di_ signals, hypothesizing that there would be a close positive relationship between RMS- and fSampEn-derived measures of oesEMG_di_.

## Experimental

### Ethical approval

This study was approved by the NHS Health Research Authority (NRES Committee London – Dulwich 05/Q0703) and the experiments conformed to the standards of the Declaration of Helsinki. All subjects were fully informed of any risk associated with the study and provided their written consent before participation.

### Subjects

Adult subjects, familiar with physiological studies, with no history of cardiorespiratory or neuromuscular disease were recruited.

### Measurements

Both invasive and non-invasive measurements of inspiratory muscle force and activation were obtained simultaneously from all subjects. Unless specified, all measures were recorded continuously during all stages of the protocol.

#### Invasive measurements

P_di_ was measured as the difference between gastric and oesophageal pressure obtained using a dual-pressure transducer tipped catheter (CTO-2; Gaeltec Devices Ltd., Dunvegan, UK) and associated amplifier (S7d; Gaeltec Devices Ltd., Dunvegan, UK), as previously described^18–20^.

Crural oesEMG_di_ was recorded using a multipair oesophageal electrode catheter (Yinghui Medical Equipment Technology Co. Ltd., Guangzhou, China). The catheter consisted of nine consecutive recording electrode coils, which formed five pairs of electrodes^2,21^. The pressure transducer and electrode catheters were inserted transnasally and once correctly positioned, taped to the nose to prevent movement during the study.

#### Non-invasive measurements

sMMG_lic_ was recorded using two triaxial accelerometers (TSD109C2; BIOPAC Systems Inc., CA, USA), and associated interface (HLT100C) and isolated power supply (IPS100C) modules (BIOPAC Systems Inc., CA, USA). The accelerometers were attached bilaterally to the skin with adhesive rings as close as possible to the surface EMG electrodes along the seventh or eighth intercostal space, over the anterior axillary line^14^.

sEMG_lic_ was recorded bilaterally using two pairs of disposable surface Ag/AgCl electrodes (H124SG; Covidien Kendall) placed on the skin over the seventh or eighth intercostal spaces, between the mid-axillary and the anterior axillary lines^8,12,17,22^. Electrode pairs were spaced 2 cm apart and a ground electrode was placed on the clavicle. The skin was appropriately prepared prior to electrode application.

Respiratory airflow was measured using a pneumotachograph (4830; Hans Rudolph Inc., KS, USA) connected to a differential pressure transducer (DP45; Validyne Engineering, CA, USA) and amplifier (CD72; Validyne Engineering, CA, USA). Mouth pressure (P_mo_) was measured from a side port on the pneumotachograph using a second differential pressure transducer (MP45; Validyne Engineering, CA, USA) attached to the amplifier.

#### Signal Acquisition

The oesEMG_di_ and sEMG_lic_ signals were amplified (gain 100), high-pass filtered at 10 Hz, and AC-coupled before acquisition (CED 1902; Cambridge Electronic Design Limited, Cambridge, UK). All signals were acquired using a 16-bit analogue-to-digital converter (PowerLab 16/35; ADInstruments Ltd., Oxford, UK) and displayed on a laptop computer running LabChart software (Version 7.2, ADInstruments Pty, Colorado Springs, USA) with analogue to digital sampling at 100 Hz (flow and pressures), 2000 Hz (sMMG_lic_), and 4000 Hz (oesEMG_di_ and sEMG_lic_).

### Maximal volitional manoeuvres

Three maximal volitional inspiratory manoeuvres were performed initially: maximal static inspiratory pressure from functional residual capacity^3^ (PImax), maximal sniff pressure, and maximal inspiration to total lung capacity^2,9^. These manoeuvres were performed sitting upright in a chair with a noseclip in place (except for maximal sniffs) and were repeated several times to ensure maximal volitional effort. P_di_, oesEMG_di_, sEMG_lic_, and sMMG_lic_ were recorded continuously during the manoeuvres and peak values determined for subsequent normalization of oesEMG_di_ recorded during the inspiratory loading protocol (see below). Each participant’s PImax value was used to determine the inspiratory threshold loads used in their individual incremental inspiratory threshold loading protocol.

### Inspiratory threshold loading protocol

All participants performed an inspiratory threshold loading protocol at five inspiratory threshold loads set at 12% (L1), 24% (L2), 36% (L3), 48% (L4), and 60% (L5) of the subject’s PImax. Inspiratory threshold loads were generated using an electronic inspiratory muscle trainer (POWERbreathe K5; POWERbreathe International Ltd., Southam, UK) attached to the distal end of the pneumotachograph. The inspiratory muscle trainer had an electronically controlled resistance valve that provided a pressure threshold resistance, which was set using the associated software (Breathe-Link, POWERbreathe International Ltd., Southam, UK). Subjects were seated and breathed through the pneumotachograph via a mouthpiece with a noseclip in place. Baseline measurements were recorded during a minimum of 2 minutes of quiet tidal breathing, following which the inspiratory muscle trainer was attached to the pneumotachograph and the series of threshold loads was imposed. Subjects were not provided with any specific instructions to adopt a certain duty cycle, and were free to choose their own breathing frequency. Subjects were, however, informed that effort was needed to overcome the threshold loads, and they were therefore encouraged to focus on using their diaphragm, to perform quick deep inspirations and to ensure that expiration was complete before making their next inspiratory effort. Each load consisted of 30 breaths followed by a resting period to allow all objective and subjective respiratory measures to return to baseline. Participants were asked to score breathlessness intensity at the end of each load using the modified Borg scale (mBorg)^23^. Participants were coached to anchor responses to mBorg 0 (no breathlessness), mBorg 10 (maximum breathlessness intensity imaginable) and mBorg 5 (severe, half maximal).

### Data analysis

LabChart data were exported as Matlab files, and analysed offline in the widely available Matlab R2014a software. All signal processing and data analysis procedures described below were automated.

#### Signal filtering and pre-processing

sMMG_lic_ signals were resampled at 200 Hz and filtered with a 4th-order zero-phase Butterworth band-pass filter between 5 and 35 Hz. Each accelerometer simultaneously provided three sMMG_lic_ signals (sMMG_lic_ X, sMMG_lic_ Y, and sMMG_lic_ Z), representing acceleration of muscle fibre vibration along all three spatial directions. A new signal, representing the total acceleration of muscle fibre vibration measured by each accelerometer, was arithmetically calculated as the norm of the vector formed by the three sMMG_lic_ signals, as follows:

$$|sMM{G}_{{\rm{lic}}}|=\sqrt{sMM{G}_{{\rm{lic}}}\,{X}^{2}+sMM{G}_{{\rm{lic}}}\,{Y}^{2}+sMM{G}_{{\rm{lic}}}\,{Z}^{2}}$$

oesEMG_di_ and sEMG_lic_ signals were resampled at 2000 Hz, and filtered with a 4th-order zero-phase Butterworth band-pass filter between 5 and 400 Hz and with four 10th-order zero-phase notch filters to remove the power line interference at 50 Hz and its harmonics at 150, 250, and 350 Hz.

#### Respiratory cycle segmentation and selection

Flow and pressure signals were segmented into inspiratory and expiratory segments by means of a zero-crossing detector on the flow signal, as previously described^24^. After segmentation all cycles were visually inspected and those either containing artefacts within the EMG and MMG signals or having an unusual P_di_ pattern were rejected. The following parameters were then calculated for each respiratory cycle: inspiratory time, total breath time, and mean P_di_. The median values of all respiratory cycles during resting breathing and threshold loading were then calculated and 10 cycles that contained the three parameters nearest to the median values were automatically selected, resulting in a total of sixty respiratory cycles for each subject.

#### P_di_ parameters

The level of inspiratory muscle force during each respiratory cycle was calculated as the mean of the inspiratory P_di_ signal. Transdiaphragmatic pressure-time product (PTP_di_), the time-integral of P_di_^25,26^, was also calculated for each respiratory cycle by multiplying the area under the curve of the inspiratory P_di_ signal by the respiratory frequency, and had units of cmH_2_O·s·min^−1^ ^26^. Both mean P_di_ and PTP_di_ parameters were calculated after removal of the baseline from the inspiratory P_di_ signal, which was determined for each respiratory cycle as the minimum level observed from the start of inspiration to the start of expiration (i.e. between points of zero flow).

#### Quantification of oesEMG_di_ signals using RMS

An additional 4th-order zero-phase Butterworth high-pass filter at 20 Hz was applied to oesEMG_di_ signals in order to reduce the P and T waves of electrocardiographic artefacts, and the low-frequency, large amplitude deflections in signal baseline produced by electrode motion and oesophageal peristalsis^27^. oesEMG_di_ signals were converted to RMS using a moving window of 50 ms with a one-sample step. The RMS peak values of oesEMG_di_ of a subject’s sixty respiratory cycles were then determined by manually analysing inspiratory oesEMG_di_ signal segments falling between QRS complexes of the electrocardiographic noise^2^. For each respiratory cycle, the highest value obtained across all five bipolar electrode pairs was selected (peak RMS oesEMG_di_). As previously described^2,9^, the per-breath RMS peak values of oesEMG_di_ were expressed as percentages of the largest RMS peak value of oesEMG_di_ obtained throughout the inspiratory threshold loading protocol and the three maximal volitional manoeuvres (peak RMS oesEMG_di%max_).

#### Quantification of EMG and MMG signals using fSampEn

Sample entropy is a measure of the complexity of a time-series signal^28^, and depends on the regularity of a signal, so that the higher the regularity, the lower the complexity, and the lower the entropy of a signal. For a given signal, regularity is calculated as the probability given by the ratio *A*/*B*, where *A* and *B* are the number of pairs of signal segments of length *m* + 1 and *m*, respectively, that are similar, that is with a maximum sample-by-sample difference less than a predefined tolerance parameter (*r*). The input parameter *m* is commonly set at 2 samples. However, *r* is usually expressed as a function of the standard deviation of the signal analysed. In fSampEn, the sample entropy is calculated within a moving window, instead of over the whole signal, using a fixed *r* value^13^. In this way, the fSampEn of a signal is a time-series whose values are higher not only when the signal is more complex, but also when the signal includes a wider range of amplitudes.

In this study, the oesEMG_di_, sEMG_lic_, and |sMMG_lic_| signals were converted to fSampEn using a moving window of 750 ms with a 50-ms step and *m* = 2. The tolerance parameter, *r*, was set at 0.1 and 0.5 times the mean standard deviation EMG and MMG values, respectively, of respiratory cycles of the upper half loads. Values of fSampEn parameters were selected in accordance with the guidelines proposed by Estrada *et al*.^29^.

The level of inspiratory muscle force and activation during each respiratory cycle was calculated as the mean inspiratory fSampEn of the MMG and EMG signals, respectively.

Analogous to PTP_di_, a novel index, the “entropy-time product (ETP)”, was calculated by multiplying the area under the curve of the inspiratory fSampEn of the MMG (ETP |sMMG_lic_|) signals by the respiratory frequency. Thus, ETP had units of s·min^−1^.

### Statistical analysis

All data are expressed as median (interquartile range). Correlation coefficients were determined to measure the relationships between the recorded signals. Normality of all the parameters calculated for each subject was tested using Lilliefors tests. Since not all parameters had a normal distribution, and a linear relationship between them could not be assumed a priori, the degree of association between parameters was measured using Spearman’s rank correlation (*ρ*). The significance level for all correlations was set at 0.05. Statistical differences in breathing pattern (inspiratory time and respiratory rate), pressures (P_mo_ and P_di_) and breathlessness (mBorg) between first and last loads of the loading protocol were analysed using non-parametric Wilcoxon signed rank tests.

Within-subject correlation coefficients were calculated over the 60 respiratory cycles of each individual. A group mean correlation coefficient of the 12 participants was also calculated for each pair of parameters using the Fisher z-transform. After applying the Fisher z-transform to the correlation coefficients of the 12 participants, the transformed z-values were averaged, and the inverse Fisher z-transform was applied to the average z-value to convert it back to a group mean correlation coefficient^30,31^.

The strength of correlation coefficients was interpreted according to Evans’ classification^32^, where correlation coefficients between 0.2 and 0.39 represent a weak correlation, coefficients between 0.4 and 0.59 represent a moderate correlation, coefficients between 0.6 and 0.79 represent a strong correlation, and coefficients of 0.8 and above represent a very strong correlation.

## Results

Twelve subjects (6 male, age 33 (30–38) years, BMI 22.2 (20.6–24.2) kg/m^2^, FEV_1_ 98.0 (94.8–105.5) % of predicted, FVC 105.0 (91.5–110.2) % of predicted, FEV_1_/FVC 82.0 (74.1–83.9) %) were recruited and completed the incremental inspiratory loading protocol.

### Breathing pattern, pressure generation and breathlessness intensity during incremental inspiratory threshold loading

**Figure 1:**
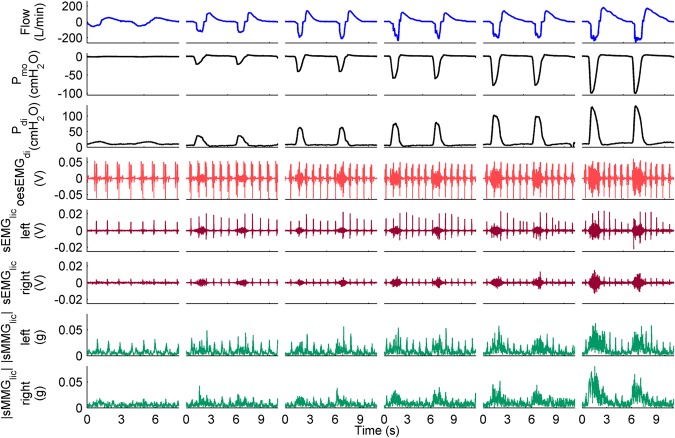
Measurements recorded during the inspiratory threshold loading protocol in a healthy subject. Two respiratory cycles are shown for quiet resting breathing and inspiratory threshold loads equivalent to 12%, 24%, 36%, 48%, and 60% of PImax (left to right). The oesEMG_di_ signal corresponds to the electrode pair 1.

Representative recordings from a single subject during resting breathing and loads 1 to 5 of the inspiratory threshold loading protocol are shown in Fig. 1.

The group median (interquartile range) PImax was 87.0 (78.0–116.5) cmH_2_O equivalent to 109.0 (89.5–126.5) % of predicted values^33^. The inspiratory threshold loads increased from 11 (10–14) cmH_2_O during load 1 to 52 (47–70) cmH_2_O during load 5. P_mo_ increased from 0.3 (0.3–0.4) cmH_2_O during resting breathing to 53.7 (49.5–68.4) cmH_2_O (*P* < 0.001) at the end of load 5. P_di_ increased from 11.1 (7.1–11.7) cmH_2_O at rest to 64.4 (46.1–67.9) cmH_2_O (*P* < 0.001) at the end of load 5. Inspiratory time ranged from 1.7 (1.4–2.1) to 2.2 (1.8–2.6) s (*P* = 0.11), and respiratory rate ranged from 13 (11–15) to 12 (10–13) breaths.min^−1^ (*P* = 0.27). mBorg breathlessness intensity increased from 0.5 (0.0–0.8) during load 1 to 4.0 (2.5–5.5) by the end of load 5 (*P* < 0.001).

### Comparison between RMS and fSampEn for myographic signal analysis

**Figure 2:**
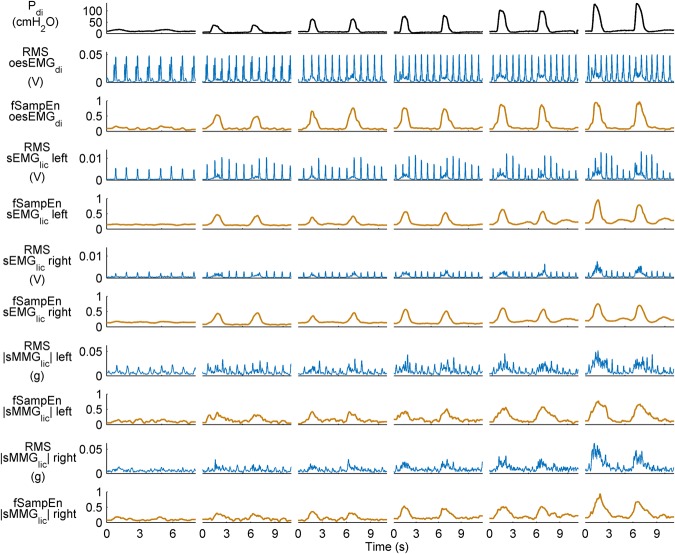
RMS and fSampEn time-series of EMG and MMG signals shown in Fig. 1. Two respiratory cycles are shown for quiet breathing and 12%, 24%, 36%, 48%, and 60% of PImax (left to right).

RMS and fSampEn time-series of a representative subject’s myographic signals are shown in Fig. 2.

**Figure 3:**
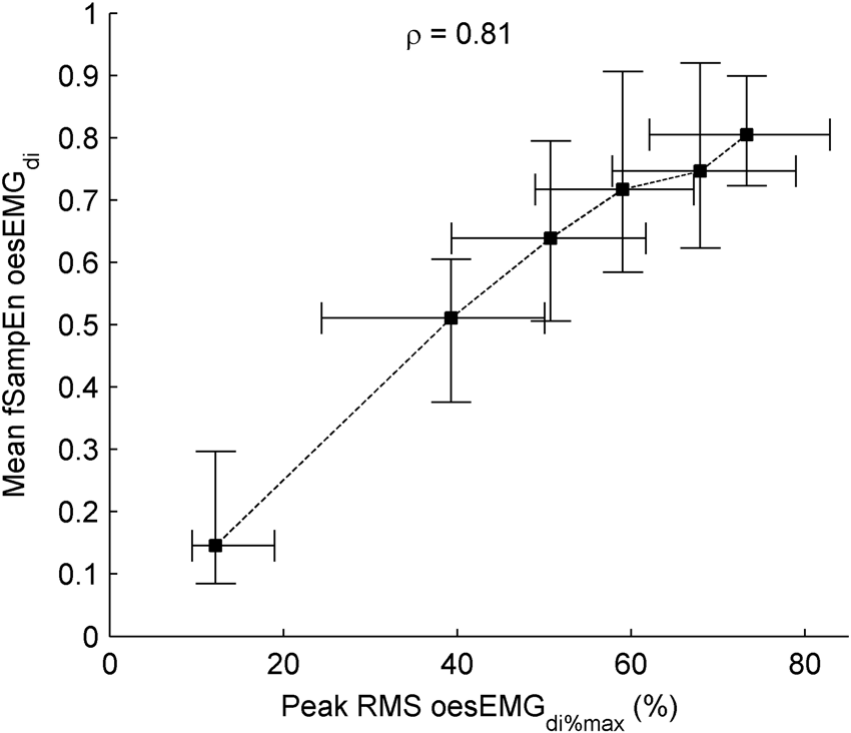
Relationship between RMS- and fSampEn-derived measures of oesEMGdi. Data points represent median and interquartile range of the 120 respiratory cycles of the twelve study subjects for each load. The group mean correlation coefficient, *ρ*, of the twelve subjects was calculated using the Fisher z-transform. Dashed lines show the order of execution of the inspiratory threshold loads.

There was a very strong positive group mean correlation between mean fSampEn oesEMG_di_ and oesEMG_di%max_ (*ρ* = 0.81) (Fig. 3). Individual correlation coefficients are shown in Table 1. All correlations were statistically significant (*P* < 0.05).

**Table 1:** Spearman’s rank correlation coefficients (*ρ*) between oesEMG_di%max_ and mean fSampEn oesEMG_di_.

Subject ID	Spearman’s *ρ* oesEMG_di%max_ – mean fSampEn oesEMG_di_
1	0.67
2	0.87
3	0.83
4	0.72
5	0.85
6	0.76
7	0.76
8	0.79
9	0.84
10	0.85
11	0.67
12	0.93
Group mean	0.81

All correlations were statistically significant (*P* < 0.05).

The RMS peaks corresponding to cardiac noise were clearly identifiable and greater in the RMS than in the fSampEn time-series (Fig. 2). This was quantified by calculating the level of cardiac noise as the average ratio, in decibels, between the peak value of segments with and without cardiac noise. This ratio was calculated for the expiratory phases of resting breathing and the final load of all RMS and fSampEn time-series shown in Fig. 2 (Table 2).

**Table 2:** Level of cardiac noise, in decibels, of the RMS and fSampEn time-series shown in Fig. 2.

	oesEMG_di_	sEMG_lic_ left	sEMG_lic_ right	|sMMG_lic_| left	|sMMG_lic_| right
RMS at rest	27.16	21.36	13.07	6.56	0.98
RMS at load 5	23.18	20.06	13.11	9.09	3.60
fSampEn at rest	4.05	0.06	0.26	2.20	−0.25
fSampEn at load 5	0.52	−0.52	−0.33	1.06	1.25

The average ratio between the peak value of segments with and without cardiac noise was calculated for the expiratory phases.

In light of the strong positive group mean correlation between mean fSampEn oesEMG_di_ and oesEMG_di%max_, and the robustness of fSampEn to cardiac noise, from here on EMG and MMG data will be presented as the mean fSampEn value only.

### Patterns of inspiratory muscle activation during incremental inspiratory threshold loading

**Figure 4:**
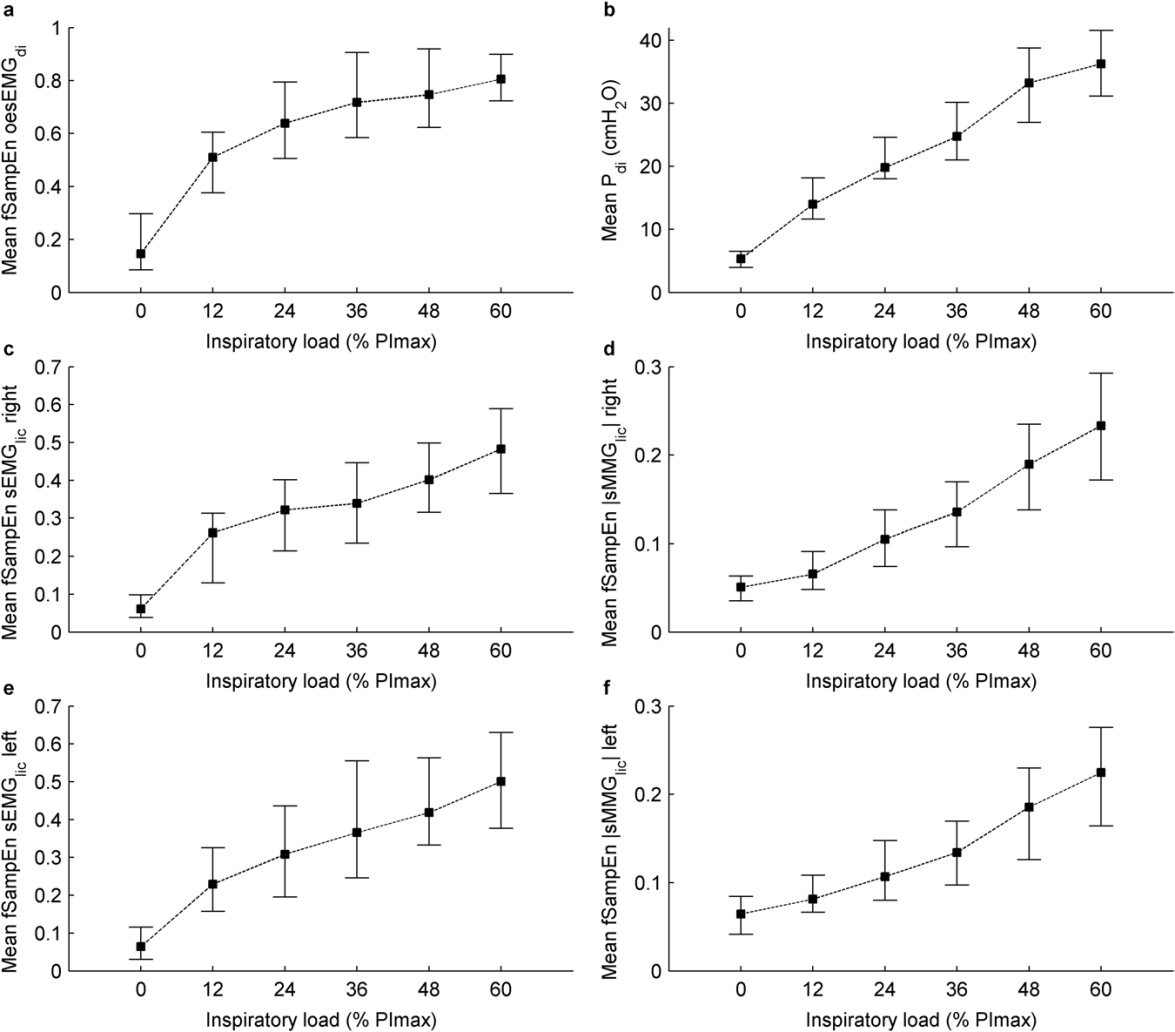
Measures of inspiratory muscle force and activation during inspiratory threshold loading. Data points represent median and interquartile range of the 120 respiratory cycles of the twelve study subjects for each load. Dashed lines show the order of execution of the inspiratory threshold loads.

In general, mean P_di_, mean fSampEn sEMG_lic_, and mean fSampEn |sMMG_lic_| increased progressively during the inspiratory threshold loading protocol. However, mean fSampEn oesEMG_di_ increased relatively less at higher than at lower loads (Fig. 4).

Mean fSampEn sEMG_lic_ behaved similarly to mean fSampEn oesEMG_di_ up to 36% of PImax, but continued to increase at inspiratory threshold loads above this level.

### Correlations between invasive and non-invasive indices of inspiratory muscle force

**Figure 5:**
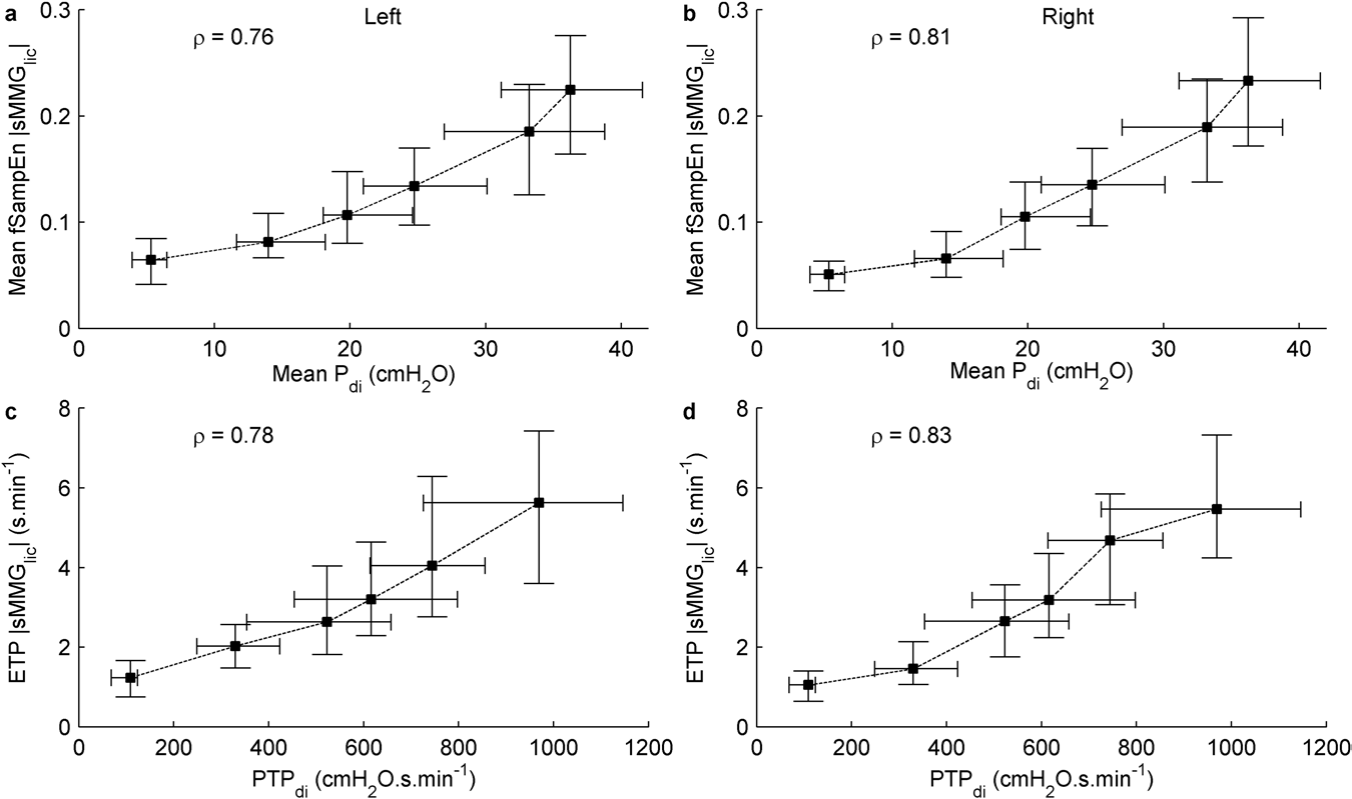
Relationship between invasive and non-invasive measures of inspiratory muscle force output recorded from the left and right sides, calculated as the mean (**a**,**b**) and time-integral (**c**,**d**) of the P_di_ and fSampEn |sMMG_lic_| signals. Data points represent median and interquartile range of the 120 respiratory cycles of the twelve study subjects for each load. The group mean correlation coefficients, *ρ*, of the twelve subjects were calculated using the Fisher z-transform. Dashed lines show the order of execution of the inspiratory threshold loads.

Strong to very strong positive group mean correlations were obtained between mean P_di_ and mean fSampEn |sMMG_lic_| left (*ρ* = 0.76, Fig. 5a) and right (*ρ* = 0.81, Fig. 5b), and between PTP_di_ and ETP |sMMG_lic_| left (*ρ* = 0.78, Fig. 5c) and right (*ρ* = 0.83, Fig. 5d). Individual correlation coefficients are shown in Table 3, and individual relationships between mean P_di_ and mean fSampEn |sMMG_lic_|, and between PTP_di_ and ETP |sMMG_lic_| are shown in the Supplementary Fig. S1. Individual and group mean correlations between the time-integrals of P_di_ and fSampEn |sMMG_lic_| signals are shown in the Supplementary Table S1. All correlations were statistically significant (*P* < 0.05). These strong positive group mean correlations were observed despite an increase in the slope of the relationship between mean fSampEn |sMMG_lic_| and mean P_di_ values at loads L2–L5 relative to loads L0–L2 (Fig. 5a,b, Supplementary Table S2).

**Table 3:** Spearman’s rank correlation coefficients (*ρ*) between invasive (P_di_) and non-invasive (sMMG_lic_) measures of inspiratory muscle force.

Subject ID	Spearman’s *ρ* Mean P_di_ – Mean fSampEn |sMMG_lic_|		Spearman’s *ρ* PTP_di_ – ETP |sMMG_lic_|	
	Left	Right	Left	Right
1	0.8	0.88	0.84	0.88
2	0.67	0.87	0.72	0.89
3	0.8	0.78	0.85	0.84
4	0.77	0.79	0.91	0.94
5	0.87	0.9	0.83	0.81
6	0.78	0.72	0.8	0.77
7	0.76	0.9	0.83	0.92
8	0.88	0.82	0.8	0.79
9	0.42	0.57	0.41	0.48
10	0.88	0.81	0.85	0.79
11	0.73	0.75	0.77	0.85
12	0.51	0.72	0.33	0.64
Group mean	0.76	0.81	0.78	0.83

Correlations between mean values, and between the corresponding time-integrals (PTP_di_ and ETP |sMMG_lic_|), are shown. All correlations were statistically significant (*P* < 0.05).

### Correlations between invasive oesEMG_di_ and non-invasive sEMG_lic_ measures

**Figure 6:**
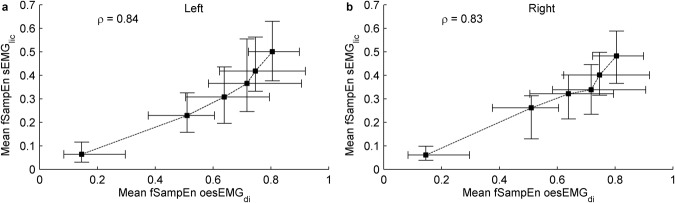
Relationship between invasive and non-invasive measures of inspiratory muscle electrical activation recorded from the left (**a**) and right (**b**) sides, calculated as the mean of the fSampEn oesEMG_di_ and sEMG_lic_ signals. Data points represent median and interquartile range of the 120 respiratory cycles of the twelve study subjects for each load. The group mean correlation coefficients, *ρ*, of the twelve subjects were calculated using the Fisher z-transform. Dashed lines show the order of execution of the inspiratory threshold loads.

Very strong positive group mean correlations were obtained between mean fSampEn oesEMG_di_ and mean fSampEn sEMG_lic_ left (*ρ* = 0.84, Fig. 6a) and right (*ρ* = 0.83, Fig. 6b). Individual correlation coefficients are shown in Table 4. All correlations were statistically significant (*P* < 0.05).

**Table 4:** Spearman’s rank correlation coefficients (*ρ*) between invasive (oesEMG_di_) and non-invasive (sEMG_lic_) signals.

Subject ID	Spearman’s *ρ* Mean fSampEn oesEMG_di_ – Mean fSampEn sEMG_lic_	
	Left	Right
1	0.82	0.83
2	0.87	0.89
3	0.82	0.82
4	0.79	0.81
5	0.82	0.87
6	0.89	0.89
7	0.76	0.67
8	0.86	0.83
9	0.87	0.9
10	0.83	0.84
11	0.85	0.79
12	0.88	0.71
Group mean	0.84	0.83

Correlations between mean values are shown. All correlations were statistically significant (*P* < 0.05).

## Discussion

In this study, sMMG_lic_, P_di_, sEMG_lic_ and oesEMG_di_ have been measured simultaneously for the first time, allowing investigation of the relationship between non-invasive and invasive indices of inspiratory muscle force, and electrical activation, respectively. The main finding of this study was the observation of strong to very strong positive correlations between sMMG_lic_, a novel non-invasive index of inspiratory muscle force output, and the invasive gold standard measure of diaphragm pressure generation, P_di_. Very strong correlations were also observed between non-invasive sEMG_lic_ and invasive oesEMG_di_ measures. Furthermore, this study is the first to demonstrate strong correlations between RMS- and fSampEn-derived measures of oesEMG_di_, and, importantly, the superior signal-to-noise ratio of fSampEn over RMS analysis of oesEMG_di_ signals, including attenuation of the cardiac artefact. This supports the use of the fSampEn technique to analyse respiratory muscle electromyogram signals.

The peak force and power output of skeletal muscle depends upon numerous factors, including muscle and fibre size and length, muscle fibre type, force-velocity relationship and the force-frequency relationship^34^. All of these factors are relevant to respiratory muscle function^7,35–38^. It is not possible to measure the force produced by the diaphragm muscle directly, and the best index of this force is P_di_, which can be recorded either during voluntary manoeuvres^39,40^ or in response to phrenic nerve stimulation^3,40,41^. P_di_ is defined as the difference between intrapleural and abdominal pressure^42^ and, in practice, is generally equated to the arithmetic difference between gastric and oesophageal pressure^3^. Measurement of P_di_ therefore requires insertion of balloon-catheters^7,41^ or a solid-state dual-pressure transducer tipped catheter^20^, as used in the present study.

The strong relationship between sMMG_lic_ and P_di_ in the present study suggests that sMMG_lic_ could provide a reliable non-invasive index of inspiratory muscle force output. The MMG signal recorded at the skin surface records the summation of the mechanical activity of single MUs as pressure waves generated by the active muscle fibres, and does not, therefore, provide a direct measure of muscle force. However, the pressure waves generated by muscle fibre activity have been suggested to reflect the mechanical aspects of muscle contraction: the gross lateral movement related to the overall change of muscle geometry at the beginning of contraction, the smaller subsequent vibrations at the resonance frequency of the muscle, and the dimensional changes in the active muscle fibres^43^. As such, sMMG has been extensively evaluated in non-respiratory muscle groups as a tool to investigate MU recruitment strategies^44,45^, and as indirect indices of muscle function, including muscle force output and fatigability^46^. Studies of locomotor^47,48^, upper limb^49–51^ and intrinsic hand muscle^50,52^ function, have reported curvilinear relationships between the RMS sMMG and force output expressed as a % of maximum voluntary contraction (%MVC). sMMG has been observed to plateau, or even subsequently decrease, at higher activation levels in a manner that differs from muscle to muscle. Since the amplitude of MMG signals is related to both the tension increase and the velocity of tension increase during twitch and tetanic contractions^53^, it has been postulated that a decrease in MMG amplitude at higher firing rates reflects fusion of the mechanical activity of MUs in relation to the elevated firing rate^50,53^. It has therefore been proposed that the variation in RMS-sMMG/%MVC relationships between muscles corresponds to differences in MU activation strategy (MU recruitment vs frequency modulation) due to differences in muscle size and histochemical type^50^. RMS-sMMG/%MVC relationships have also been reported to vary with age^49^, sex^51^, and joint angle^54^. Single MU recordings of the diaphragm have previously shown that the diaphragm muscle predominantly employs a strategy of frequency modulation, rather than progressive recruitment of MUs throughout contraction^55^. In this way, the behaviour of the diaphragm is, interestingly, more similar to that of intrinsic hand muscles than that of large proximal muscles^56^. RMS sMMG recordings contributed to by diaphragm muscle fibres alone would therefore be expected to plateau at a relatively low %PImax, akin to RMS-sMMG/%MVC relationships observed in intrinsic hand muscles^50^. In the present study, however, both mean fSampEn sEMG_lic_ and mean fSampEn |sMMG_lic_|, but not mean fSampEn oesEMG_di_, increased progressively as %PImax and P_di_ increased, without an observable plateau. The lack of progressive increase in mean fSampEn oesEMG_di_ likely reflects the increasing contribution of extradiaphragmatic muscle activity to non-invasive |sMMG_lic_| and sEMG_lic_ signals, but not to invasive oesEMG_di_ signals, recorded at the highest inspiratory threshold loads^57^.

Few previous studies have investigated the utility of sMMG in respiratory muscle physiology. Petitjean and Bellemare^12^, using condenser microphones, reported significant linear relationships between the amplitude of the right and left phonomyogram and P_di_ peak values, elicited by phrenic nerve stimulation. Recently, Sarlabous *et al*.^15^ used uniaxial accelerometers to record sMMG_lic_ in patients with chronic obstructive pulmonary disease and healthy subjects during an incremental inspiratory flow protocol. Very strong correlations were observed between sMMG_lic_ and peak inspiratory P_mo_. There was also a strong correlation between sMMG_lic_ and FEV_1_ in patients, suggesting the potential utility of sMMG_lic_ as a novel non-invasive index of the mechanical load on the respiratory muscles. Distinct to this previous work, the present study employed triaxial accelerometers to record sMMG_lic_, allowing inspiratory muscle vibrations to be measured in all three spatial directions. Uniaxial accelerometers detect muscle mechanical activity in one spatial direction only and can therefore lead to an underestimation of muscle mechanical activation. Moreover, inspiratory threshold loading allowed the sMMG_lic_ to be measured over a wider range of respiratory effort than the inspiratory flow protocol employed previously by Sarlabous *et al*.^15^.

Invasive measurement of P_di_ and oesEMG_di_ is technically complex, requires some skill from the operator involved, may be time-consuming, and most importantly can be uncomfortable for study participants. In this regard, non-invasive techniques would facilitate the assessment of inspiratory muscle force and activation in physiological, and clinical, studies at scale. In contrast to sMMG, sEMG has been used extensively to assess respiratory muscle activation^17,58–62^. Reilly *et al*.^20^ reported a very strong correlation between non-invasive parasternal intercostal muscle EMG (sEMG_para_) and oesEMG_di_ during an incremental cycle exercise test to exhaustion in healthy subjects and in cystic fibrosis patients with chronic airway obstruction. PTP_di_ increased with increasing sEMG_para_ in healthy subjects, whereas PTP_di_ in cystic fibrosis patients plateaued at submaximal oesEMG_di_ and sEMG_para_ levels reflecting neuromechanical uncoupling as a consequence of impaired respiratory mechanics in chronic lung disease. Simultaneous recordings of sEMG_lic_ and sMMG_lic_, as described in the present study, could therefore provide the potential to assess the relationships between electrical and mechanical activation of inspiratory muscles in a wholly non-invasive manner. The oesEMG_di_ is increasingly recognized to provide an index of neural respiratory drive which, together with measures of respiratory muscle pressure generation, are facilitating a greater understanding of the neurophysiology of breathlessness perception in health and in respiratory disease^10,63,64^. sMMG_lic_ and sEMG_lic_ could therefore provide non-invasive estimates of neural respiratory drive to further this work at scale. A combination of sMMG_lic_ and sEMG_lic_ could also provide clinically applicable non-invasive indices of respiratory muscle function that could be particularly useful in the following settings: monitoring of acute exacerbations of chronic obstructive pulmonary disease^65,66^, weaning from mechanical ventilation in critical care settings^67^, monitoring of diaphragm weakness as a prognostic factor in motor neuron disease^1^, and monitoring patients who cannot reliably perform lung function^68^. The increase in the slope of the relationship between the non-invasive measures (sMMG_lic_ and sEMG_lic_) and invasive measures (P_di_ and oesEMG_di_) at the highest inspiratory threshold loads, which we suggest is due to increased extradiaphragmatic respiratory muscle activation, occurred at relatively high levels of diaphragm activation (median(IQR) oesEMG_di%max_ was 50.7% (39.3%-61.7% at load L2). This level of oesEMG_di%max_ is higher than the values typically recorded at rest even in severe COPD patients^2^, and approaches end-exercise values in health and in respiratory disease^10,20,64^. This change in slope is therefore unlikely to significantly limit the utility of these non-invasive correlates of inspiratory muscle activation within typical clinical settings.

A major novel feature of this study is the use of the fSampEn to analyse the simultaneously recorded oesEMG_di_, sEMG_lic_, and sMMG_lic_ signals. The fSampEn of a signal is a time-series whose values not only depend on the signal amplitude, but also on the signal complexity. Like RMS, fSampEn can track amplitude changes evoked by EMG and MMG activity. However, since cardiac artefacts are much more regular (less complex) than EMG and MMG signals, which are random in nature, fSampEn is less influenced by cardiac artefacts than RMS, as previously described for sMMG_lic_^13^ and sEMG_lic_ signals^17^. In this study, the potential of fSampEn to reduce cardiac artefacts has been shown, for the first time, in oesEMG_di_ signals, which are more affected by cardiac cross-talk than sEMG_lic_ and sMMG_lic_ signals. Based on the advantageous properties of fSampEn, the entropy-time product has been proposed in this study as a new index to measure inspiratory muscle force output from sMMG_lic_ signals. The ETP parameter allows inspiratory muscle activity to be analysed over the whole inspiratory phase of a respiratory cycle regardless of cardiac noise which can be markedly elevated depending on recording site. Accordingly, no relevant differences have been found between correlation values of left and right sides in healthy subjects. However, an RMS-based analysis, which has been the conventional approach to analyse EMG signals, implies prior rejection of signal segments that contain cardiac noise, and therefore provides only coarse estimates of inspiratory muscle activity, since only the RMS peak value of the signal generated during muscle activation is analysed. Moreover, this is a subjective and time-consuming task when performed manually. Although some automatic algorithms have been previously proposed to remove cardiac noise from EMG signals^69–71^, these algorithms involve the recording of an extra electrocardiographic channel for QRS complexes detection.

Despite the potential for using sMMG_lic_ and sEMG_lic_ to assess inspiratory muscle force and activation non-invasively, we acknowledge limitations of our study, which invites further investigation. Firstly, although our findings suggest that sEMG_lic_ and sMMG_lic_ are useful indices of inspiratory muscle function, these measures are not specific for the diaphragm, since, as discussed above, we cannot exclude the contribution of extradiaphragmatic chest wall and abdominal musculature to these non-invasive signals, particularly during loaded breathing^72,73^. There is no consensus standard for optimum surface EMG electrode positioning during non-invasive assessment of diaphragm activation, with several recording sites having been suggested for recording electrode placement^8,59,74–79^. There is also no consensus on methods to maintain electrode orientation with respect to the muscle fibres or to control for the influence of variable muscle-to-electrode distance, such as subcutaneous fat or chest wall deformities that produce variable muscle-to-electrode filtering effects^3^. The participants in our study cohort were twelve healthy subjects with BMI values within the normal range and the effect of BMI in sMMG_lic_ and sEMG_lic_ measures should therefore be a focus of future research. Future work should also investigate the reproducibility of the sMMG_lic_ measures in health and disease.

Regarding fSampEn, although this parameter tracked amplitude changes of related physiological signals in this and in previous studies^14,15,17^, fSampEn values have no units and the true physical meaning remains unclear. Therefore, definition of a normal range of values and a normalization process for fSampEn, as that described by Jolley *et al*.^2^ for oesEMG_di_, is another important area of research.

In summary, we found strong correlations between non-invasive sMMG_lic_ and sEMG_lic_ measures and invasive P_di_ and oesEMG_di_ measures, respectively, in healthy subjects. The strong correlation between sMMG_lic_ and P_di_ suggests that sMMG_lic_ could provide a novel non-invasive correlate of inspiratory muscle force through which to further the study of the physiology, and pathophysiology, of inspiratory muscle function in health and disease.

## Electronic supplementary material

Supplementary Information

## Data Availability

The datasets analysed during the current study are available from the corresponding author on reasonable request.
