# Supplementary material for: Surface mechanomyography and electromyography provide non-invasive indices of inspiratory muscle force and activation in healthy subjects
Source: Sci Rep. 2018 Nov 16;8:16921. doi: 10.1038/s41598-018-35024-z (PMC6240075; doi:10.1038/s41598-018-35024-z)
Supplement: Supplementary file 1 — Supplementary Information [file 41598_2018_35024_MOESM1_ESM.pdf]

Surface mechanomyography and electromyography provide non-invasive indices of inspiratory muscle force and activation in healthy subjects

Manuel Lozano-García, Leonardo Sarlabous, John Moxham, Gerrard F Rafferty, Abel Torres, Raimon Jané, Caroline J Jolley

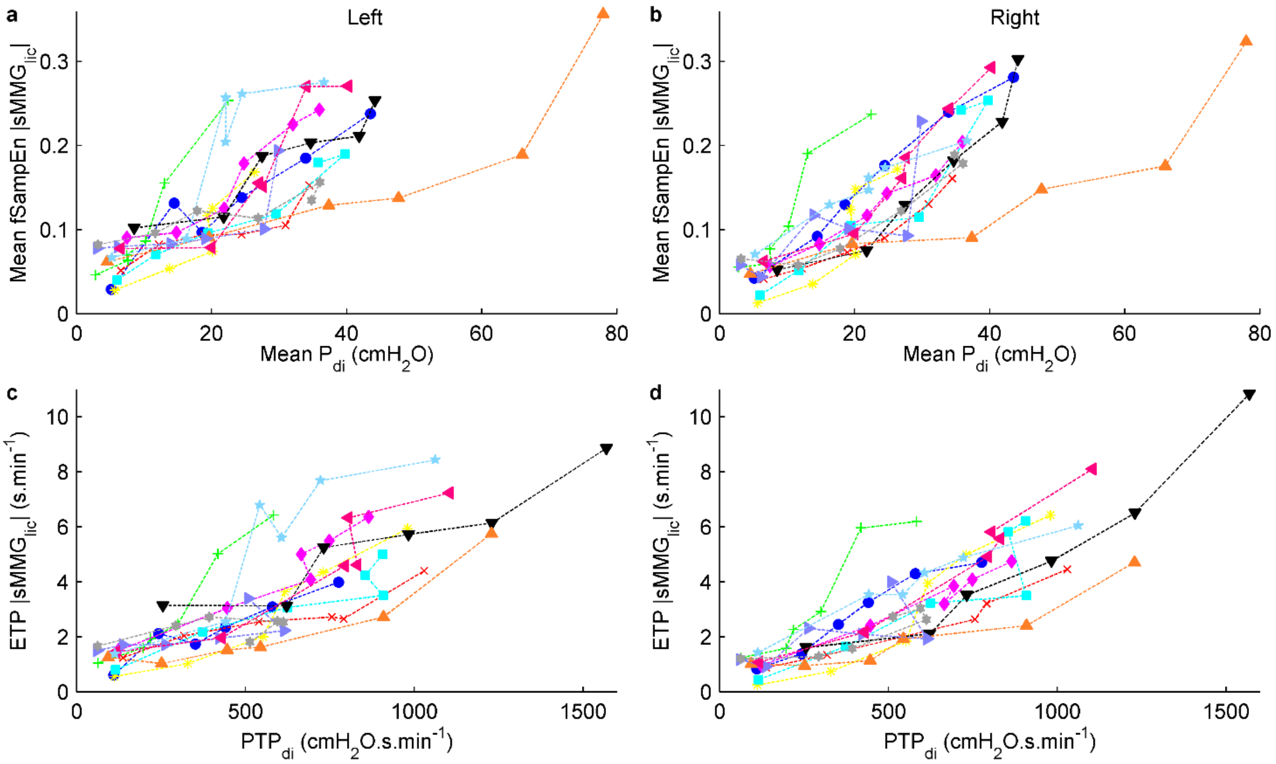

Figure S1. Individual relationships between invasive and non-invasive measures of inspiratory muscle force output recorded from the left and right sides, calculated as the mean (a, b) and time-integral (c, d) of the P_{di} and fSampEn $|sMMG_{lic}|$ signals. Data points represent median of each subject's ten respiratory cycles for each load. Dashed lines show the order of execution of the inspiratory threshold loads.

Table S1. Spearman's rank correlation coefficients (ρ) between invasive (P_{di}) and non-invasive ($sMMG_{lic}$) measures of inspiratory muscle force.

**Subject ID**	**Spearman's ρ AUC P_{di} – AUC fSampEn $\lvert sMMG_{lic}\rvert$**	
	**Left**	**Right**
1	0.86	0.89
2	0.70	0.88
3	0.81	0.79
4	0.88	0.91
5	0.82	0.85
6	0.65	0.71
7	0.89	0.95
8	0.86	0.83
9	0.47	0.57
10	0.87	0.83
11	0.73	0.87
12	0.54	0.72
**Group mean**	**0.78**	**0.84**

Correlations between the area under the curve (AUC) are shown. All correlations were statistically significant (P<0.05).

Table S2. Simple linear regression slopes and correlation coefficients between invasive (P_{di}) and non-invasive ($sMMG_{lic}$) measures of inspiratory muscle force.

Subject ID	Simple linear regression Mean P_{di} – Mean fSampEn $\|sMMG_{lic}\|$							
	Left				Right			
	L0-L2		L2-L5		L0-L2		L2-L5	
	Slope*	r	Slope*	r	Slope*	r	Slope*	r
1	0.58	0.63	0.64	0.78	0.67	0.72	0.68	0.70
2	0.39	0.70	0.18	0.35	0.29	0.72	0.44	0.63
3	0.47	0.50	1.17	0.82	0.46	0.44	0.94	0.68
4	0.30	0.69	1.02	0.59	0.40	0.87	1.16	0.51
5	0.40	0.72	0.52	0.70	0.54	0.83	0.78	0.75
6	0.21	0.41	0.64	0.55	0.39	0.84	0.35	0.38
7	0.36	0.59	0.41	0.39	0.37	0.66	0.92	0.74
8	0.17	0.63	0.47	0.76	0.12	0.46	0.42	0.72
9	0.02	0.04	0.47	0.54	0.37	0.38	0.49	0.48
10	0.42	0.69	0.99	0.75	0.40	0.67	0.77	0.55
11	0.76	0.59	0.31	0.23	0.58	0.77	0.50	0.59
12	0.17	0.42	0.21	0.35	0.12	0.34	0.51	0.56
**Median (IQR)**	**0.37 (0.20-0.43)**	**0.55 (0.48-0.69)**	**0.50 (0.39-0.73)**	**0.57 (0.38-0.75)**	**0.40 (0.35-0.48)**	**0.64 (0.45-0.79)**	**0.59 (0.48-0.82)**	**0.61 (0.54-0.70)**

* Slope values x 100. IQR: interquartile range. L0-L5 correspond to inspiratory threshold loads of 0%, 12%, 24%, 36%, 48% and 60% of each subject's PImax.
